# Are abnormalities missed in the PERFORMS self-assessment scheme due to visual or cognitive factors?

**DOI:** 10.1186/bcr3700

**Published:** 2014-11-03

**Authors:** Y Chen, L Dong, A Gale

**Affiliations:** 1 Loughborough University, Loughborough, UK

## Introduction

To examine whether missed abnormalities in PERFORMS test sets are mainly due to visual or cognitive factors.

## Methods

Data were examined from three recent rounds of the PERFORMS breast screening self-assessment scheme (that is, three sets of 120 difficult normal, benign and malignant FFDM cases) that had respectively been reported by 723, 726 and 687 breast radiologists and advanced practitioners in order to determine the error rates and the underlying reasons for such errors. Every participant had read each case set in a different random order.

## Results

The cancer detection in the three rounds was high, with mean values of 82%, 86% and 93%. However, in terms of discrepant recall decisions between individuals and overall decisions, there were both over-reading (for the normal and benign cases) and under-reading errors (for the malignant cases) for many cases; although there was no statistical difference in the error rate between these three case categories. Overall, for the normal cases there were 15.55% false positives, with the majority of these being scored as indeterminate (13.37%). For malignant cases there were 16% false negatives, with 11.45% reported normal and 4.12% benign. Further analyses demonstrated that the majority of the false negative errors were due to mammographic features being missed (unseen) rather than being seen but then misinterpreted - a cognitive error (*t *test, *P *< 0.002).

## Conclusion

In examining these difficult case sets from the PERFORMS scheme, most errors were due to key abnormal mammographic features not being seen.

